# Mesenchymal Stem Cells Inhibits Migration and Vasculogenic Mimicry in Nasopharyngeal Carcinoma *Via* Exosomal MiR-125a

**DOI:** 10.3389/fonc.2022.781979

**Published:** 2022-02-17

**Authors:** Fangzhu Wan, Haojiong Zhang, Jiyi Hu, Li Chen, Shikai Geng, Lin Kong, Jiade J. Lu

**Affiliations:** ^1^ Department of Radiation Oncology, Shanghai Proton and Heavy Ion Center, Fudan University Cancer Hospital, Shanghai, China; ^2^ Shanghai Key Laboratory of Radiation Oncology (20dz2261000), Shanghai, China; ^3^ Shanghai Engineering Research Center of Proton and Heavy Ion Radiation Therapy, Shanghai, China; ^4^ Department of Radiation Oncology, Shanghai Proton and Heavy Ion Center, Shanghai, China; ^5^ Rui-jin Hospital, Shanghai Jiao-tong University School of Medicine, Shanghai, China

**Keywords:** nasopharyngeal carcinoma, vasculogenic mimicry, mesenchymal stem cell, microRNA, exosome

## Abstract

Vasculogenic mimicry (VM) is a kind of tumor vasculature providing blood supply for tumor growth, and the formation of VM is independent of vascular endothelial cells. Instead, VM structures are formed by differentiated tumor cells such as nasopharyngeal carcinoma cells. Recently, studies have shown that anti-angiogenic therapy failed to improve the overall survival for patients, namely, nasopharyngeal carcinoma patients. The existence of VM structure is probably one of the reasons for resistance for anti-angiogenic therapy. Therefore, it is important to study the mechanism for VM formation in nasopharyngeal carcinoma. In this study, the bioinformatic analysis revealed that microRNA-125a-3p (miR-125a) was highly expressed in normal nasopharyngeal epithelial tissue than in nasopharyngeal carcinoma. An *in vitro* study demonstrated that miR-125a plays an inhibitory role in nasopharyngeal carcinoma cell migration and VM formation, and further studies confirmed that TAZ is a direct downstream target for miR-125a. On this basis, we artificially engineered human mesenchymal stem cells (MSCs) to generate exosomes with high miR-125a expression. Treatment with these miR-125a-over-expressing exosomes attenuated the migration and VM formation in nasopharyngeal carcinoma cells. In addition, the inhibitory role of these exosomes on VM formation and migration in nasopharyngeal carcinoma was also confirmed *in vivo*. Overall, the current study shows that MSCs can be utilized to generate exosomes with high miR-125a level, which could be therapeutic nanoparticles targeting VM formation in nasopharyngeal carcinoma and used as a complement to anti-angiogenic therapy in the future.

## Introduction

Nasopharyngeal carcinoma (NPC) is a unique class of head and neck malignant tumor, and is endemic in eastern parts of Asia and North Africa (1, 2). Standard treatment for NPC is radiotherapy (RT) for early-stage lesions or chemoradiotherapy for more advanced lesions. Although recent improvement in treatment has been achieved, many NPC patients still have poor prognosis, suffering distant metastases or locoregional recurrence (3), which is one of the predominant reasons for therapy failure.

Angiogenesis is a hallmark of cancer which is essential for growth, invasion, and metastases of tumors, and has long been proposed as a therapeutic target in oncology (4). Studies have focused on the angiogenesis involving endothelial cells activation and recruitment of new vessels in tumor. However, clinical trials in head and neck squamous cell carcinoma (HNSCC) show that anti-angiogenic drugs such as bevacizumab, have not showed apparent improvement in efficacy (5). In some cases the anti-angiogenic therapy is discontinued due to poor response to treatment and life-threatening side effects (6). The reason may be that the blood supply in a tumor is partially dependent on non-endothelial cell vessels.

Vasculogenic mimicry is the channel conducting blood or other liquid formed by genetically dysregulated tumor cells rather than endothelial cells (ECs). Periodic acid-Schiff (PAS) staining combining with CD34 immunohistochemistry (IHC) can be used to detect the structure of VM (7). VM has been shown to be present in various malignant tumors (8), namely, NPC (8), which is associated with tumor metastasis (9) and poor prognosis (7). Since the structure of VM is composed of cancer cells, the benefit of VEGF-targeted therapy which is focused on ECs is limited and unsatisfied. The transcriptional symbol of ‘VM Formation’ (10) shares similarities with that of ‘stemness’ and ‘Epithelial-to-Mesenchymal Transition (EMT)’, both of which are key characteristics relative to tumor development during invasion and resistance to radiation treatments or chemotherapy drugs (11). Besides, EBV infection confers neovascular features in epithelial cancers especially in NPC by promoting VM formation (8).

MicroRNAs (miRs) are small noncoding, single-stranded RNAs, which are now known to play an vital role in tumor malignancy (12, 13). MiR-125a is located on chromosomes 19, 11, and 21 and is believed to be involved in proliferation, apoptosis, migration and invasion in diverse cancers. In NPC, miR-125a was downregulated and associated with chemotherapy sensitivity (14) and radiation sensitivity (15).

Exosomes are membrane-bound vesicles which can be secreted by different cells. The diameter of exosomes is reported to be 30–150 nm. Exosomes are found to be important messengers for cell–cell communication (16) transferring molecules such as protein or nucleic acids from cells to cells. Some exosomes act as suppressor for tumor growth and thus have the potential function for tumor treatment (17). Mesenchymal stem cells (MSCs) are multipotent stromal cells (18), which have the potential to treat a variety of diseases (19), and could be used for tumor treatment (20). Moreover, large amounts of MSC-derived exosome are reported to be enriched in tumors (21). Thus, MSC-exosomes could serve as therapeutic vehicles for NPC transferring anti-tumor miRs.

In this study, we report for the first time that miR-125a inhibits invasion, migration and VM formation in NPCs *via* inhibiting TAZ. Furthermore, we confirmed that MSC-exosomes could be engineered and used as anti-tumor vehicles containing miR-125a to inhibit invasion, migration, and VM formation in NPC cells *in vivo* and *in vitro*.

## Materials and Methods

### Quantitative Real-Time PCR (qRT-PCR)

Trizol reagent (Invitrogen, USA) was used to extract the total RNA in NPC cell lines. Then the reverse transcription was continued referring to the protocol by High Capacity cDNA Reverse Transcription Kit (Biosystems). The cDNA was utilized for real-time PCR *via* Mx-3000P Quantitative PCR System (Stratagene). GAPDH and U6 were selected as control group. The following were the primer sequences for different genes: miR-125a-5p forward, 5′-GGTCA TTCCCTGAGACCCTTTAAC-3′; reverse, 5′-GTGCAGG GTCCGAGGT-3′. TAZ forward, 5′-ACCCACCCACGATGACCCCA-3′; reverse, 5′-GCACCCTAACCCCAGGCCAC-3′; GAPDH, forward, 5′-GGAGCCAAAAGGGTCATCAT-3′; reverse, 5′-GTGATGGCATGGACTGTGGT-3′. U6 forward, 5′-CTCGCTTCGGCAGCACA-3′; reverse 5′-AACGCTTCACGATTTGCGT-3′. The relative mRNA level was normalized by the expression of GAPDH, and the relative miR level normalized by expression of U6. The independent experiments were performed 3 times, each of which used 3 samples independently.

### Target Prediction

The TAZ gene was queried by the miRNA target prediction sites TargetScan (Human) (http://www.targetscan.org/), miRbase (http://www.mirbase.org/) and miRDB (http://mirdb.org/miRDB). The scores and strength of binding sites for predicted target were detected. The miRNAs overlapping in more than 2 searches were selected for further studies.

### Databases

The clinical information and also microRNA expression levels for clinical samples in NPC and HNSCC were acquired from the GEO (Gene Expression Omnibus) database (NCBI/GEO/GSE32960) and TCGA database (https://tcga-data.nci.nih.gov/tcga/). All the data were analyzed with GraphPad Prism.

### Cell Culture

NPC cell lines such as HONE-1, 6-10B, and 5-8F were maintained in our lab and cultured in RPMI 1640 medium (GIBCO) with 10% FBS, supplemented with 100 U/ml penicillin and 100 mg/ml streptomycin. The keratinocyte serum-free medium (KSFM, Invitrogen, Carlsbad, CA, USA) with human recombinant epidermal growth factor (rEGF) was used to culture the NP-69 cells and NP-460 cells which were immortalized nasopharyngeal epithelial cell line. The 293 T cells were maintained in our lab and were cultured for luciferase reporter assay.

### Exosome Preparation

MSC were cultured and the exosomes were isolated from the conditioned medium using Exosome isolation Kit—Serum and Plasma (Exiqon) according to protocol, the procedures of which were performed in previous studies (22). The electron microscopy and nanoparticle tracking technology (Nanosight™) were used for identification for exosomes.

### Luciferase Reporter Assays

The pGL3-TAZ and pGL3-mutTAZ reporter genes were both established by Bio-Asia (China). The luciferase reporters and the miR-125a mimics were both transfected in 293T cells. The activity of the reporter protein was detected by a luciferase assay kit (Promega) after transfected for 48 h with reference to the instructions.

### IHC (Immunohistochemistry) for Tissues

The paraffin-embedded tissues were sliced and heated for further investigation. After deparaffinized, rehydrated and antigen retrieving, the slides were blocked with goat serum and incubated with primary antibody (Abcam) at 25°C for 4 h. After the usage of a secondary antibody and DAB (3,3′-diaminobenzidine) substrate solution, the picture were captured. Matrix-associated vascular channels including the vasculogenic mimicry structure were detected by PAS staining. Images for all the slides were captured by Leica DM 2500 microscope.

### Assay for VM Formation

Matrigel were coated in 96 wells plates (50 μl/well), and NPC cells (6-10B and 5-8F) were seeded in the wells (3 × 10^4^ cells per well) and cultured for 18 h by RPMI 1640 medium without FBS. Images were took after 36 h using Leica DM 2500 microscope randomly.

### MiR-125a Overexpressing and Knockdown

The miR-125a mimics, miR-125a inhibitors and corresponding negative control were purchased from Genepharma (Shanghai, China). Lentiviruses expressing miR-125a, inhibiting miR-125a and the scrambled control were constructed by Genechem (Shanghai, China). The transfecting with lentivirus were performed at MOI = 10 in the NPC cells and at MOI = 10 in the human mesenchymal stem cells.

### Transwell Migration Assays

NPC cells were seeded in the top chamber cultured by serum-free media. The bottom chamber was filled with RPMI 1640 medium containing 15% FBS. After 36 h (6–10 B) or 24 h (5–8 F) of incubation, the cells were simply removed and fixed with 4% paraformaldehyde in the top chamber for 20 min, and then stained with giemsa solution for 20 min. Five fields per well were photographed at random.

### Tumour Xenograft Mouse Model

A total of six BALB/c nude mice (male, 4–5 weeks old) were purchased in the SLAC Laboratory Animals (Shanghai), and were randomly divided into 2 groups. After disinfection, stably transfected cells (1 × 107 cells/ml, 0.2 ml of cell suspension) were subcutaneously injected at the left groin. Tumor growth was detected, and the diameters of long (a) and short (b) for each tumor were measured every 3 days. Tumor volumes were calculated as V = 0.5 × a × b^2^ and then the tumor growth curves were generated.

### Western Blotting

The cultured cells were harvested using RIPA cell lysis buffer. All the protein lysates in different groups were loaded and separated by SDS-PAGE, and the SDS PAGE gel were transferred to a polyvinylidene difluoride (PVDF) membrane. The blots were incubated with primary antibodies against TAZ (Proteintech; China), LAMB2 (Proteintech; China), N-cadherin (Cell Signaling Technology; USA), E-cadherin (Cell Signaling Technology; USA), MMP2 (Cell Signaling Technology; USA), MMP9 (Cell Signaling Technology; USA), and GAPDH (Abcam; UK). The enhanced chemiluminescence (ECL, Millipore, USA) was utilized for visualizing the protein bands. The intensity of the protein bands was analysed *via* ImageJ 1.52a software (National Institutes of Health, USA) and normalized to the bands of GAPDH.

### Wound-Healing Migration Assay

NPC cells were cultured in RPMI 1640 medium containing 15% FBS in 6-well plates at 37 °C for 24 h. Wounds were generated by scratching with 200 ul sterile pipet tips, and washed 3 times with phosphate-buffered saline (PBS). After wounds were made, cells were cultured in RPMI 1640 medium containing 1% FBS. Then images were captured at the time 0 h and 36 h after wounding.

### Statistical Analysis

All statistical data were analyzed using GraphPad Prism 7.00 software. Student’s t-test was used for calculating significance between two groups, and one-way ANOVA was used for multiple comparisons. Kaplan–Meier survival curves were also analyzed *via* log-rank tests using GraphPad Prism 7.00 software. All tests were two-sided, and p-values <0.05 were considered statistically significant. All data are presented as the mean ± standard error of the mean. Bar graphs represent mean and SD or mean and SEM across three independent experiments.

## Results

### miR-125a Level is Downregulated in NPC Tissues and Cell Lines and is Associated With Patient Prognosis

**Figure 1:**
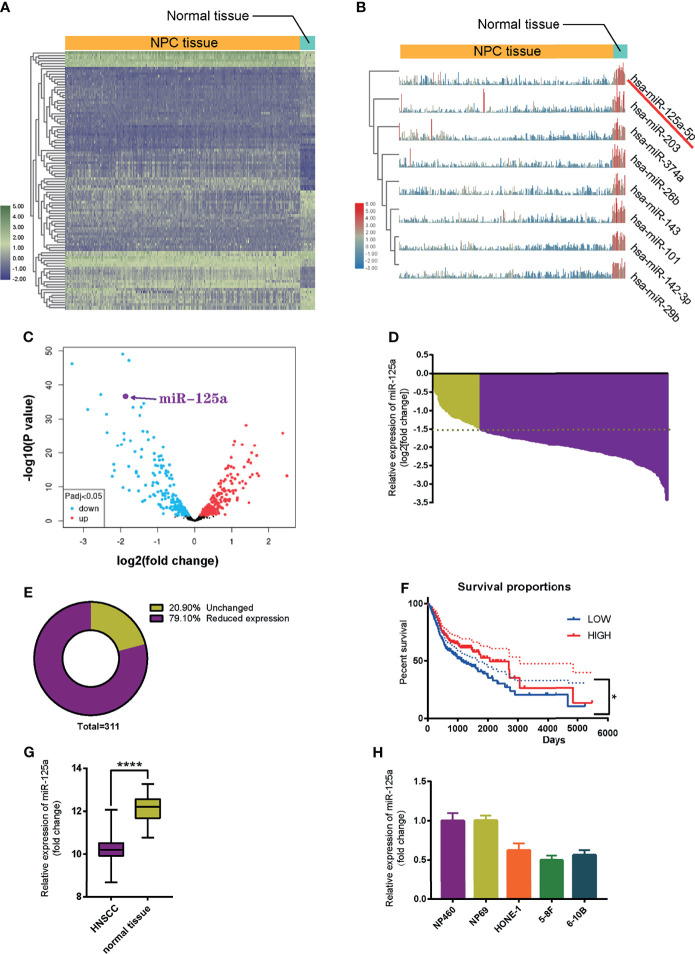
MiR-125a is downregulated in NPC tissues and cell lines. **(A)** Heat map of top 250 differentially expressed genes. **(B)** Illustration of top 8 downregulated genes. **(C)** Volcano plot of differentially regulated microRNAs. **(D)** Waterfall plot for mir-125a expression level of each NPC patient (NCBI/GEO/GSE32960). **(E)** Pie chart summarizing percentage of miR-125a level of NPC patients, a fold change >2 or <1/2 was defined as significant. **(F)** Kaplan–Meier survival curve in miR-125a low and high expression NPC cases (NCBI/GEO/GSE32960). **(G)** Box plot for miR-125a level in HNSCC and normal tissue in the TCGA database. **(E, H)** Relative mir-125a expression in normal nasopharynx cell lines and NPC cell lines. (*P < 0.05; ****P < 0.0001).

To identify miRNA candidates involved in NPC initiation and progression, we analyzed and compared microRNA expression levels between NPC tissue and normal nasopharynx tissue using the Gene Expression Omnibus (GEO) database (NCBI/GEO/GSE32960). Heatmap (23) (**Figures 1A, B**) and volcano plot (**Figure 1C**) visualization of gene expression were constructed, and we found that miR-125a significantly decreased in NPC tissue samples compared to normal nasopharynx samples. An absolute fold-change cut-off of ≥1.5 was used for statistical analysis. Indeed, miR-125a was significantly downregulated in 79.10% NPC patients (n = 311). In the rest of NPC patients, miR-125a expression level also exhibited a tendency of declination, although the differences failed to reach the statistical significance (**Figures 1D, E** **)**. In addition, patients with high expression of mir-125a had higher survival rate as well as better prognosis (**Figure 1F**). The TCGA database validated the downregulation of mir-125a in HNSCC (**Figure 1G**). Moreover, the normal nasopharynx cell lines displayed the higher miR-125a expression than NPC cell lines (**Figure 1H**).

To sum up, these results indicated that the low level of miR-125a is related to development of NPC cells.

### miR-125a-3p Inhibits Invasion, Migration and VM Formation in NPC Cells

**Figure 2:**
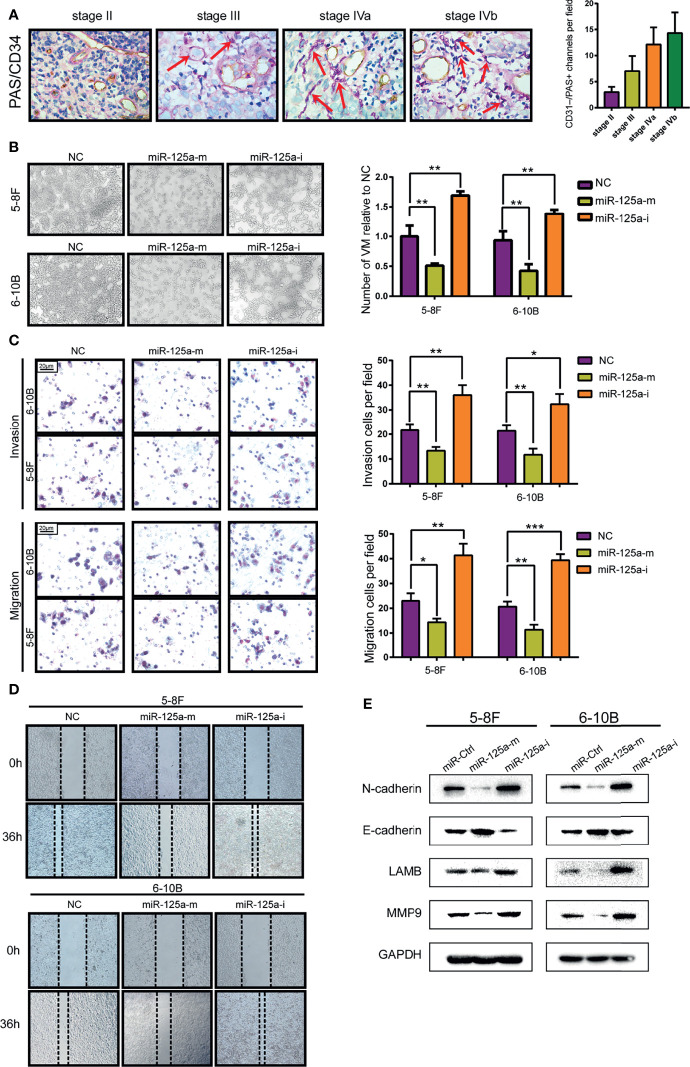
MiR-125a decreases the level of VM formation, migration and EMT level of NPC cells. **(A)** NPC tissue at different stage (AJCC TNM Staging System 2017) staining by PAS/CD34 and corresponding histogram. **(B)** VM formation assay (left panel) and bar graph showing relative number of VM (right panel). **(C)** Invasion and migration transwell assay and histogram showing cell counting per field. **(D)** Wound healing assay. **(E)** Western blot assay detecting EMT-related protein. (*P < 0.05; **P < 0.01; ***P < 0.001).

We showed VM structures in NPC tissue by double staining with PAS and CD34, in line with previous studies in other neoplasms. VM structures could be observed in NPC tissues, which were negative for vascular endothelial marker CD34 but positive for PAS (**Figure 2A**). We also found that the high graded of NPC stage is correlated with the higher level of VM structure, indicating that VM structures are related to advanced-stage and poor prognostics (**Figure 2A**). Then we conducted VM formation assay and found that over-expression of miR-125a hampered VM-forming abilities (**Figure 2B**). In contrary, inhibition of miR-125a promoted the formation of VM in NPC cells (**Figure 2B**).

As the transcriptional signature of VM is associated deeply to migration ability and Epithelial-to-Mesenchymal Transition (EMT) (24), we therefore detected the invasion and migration characteristics (**Figures 2C, D** **)** and EMT-related protein level (**Figure 2E**) of NPC cells. We found that upregulation of miR-125a decreased the invasion and migration ability of NPC cells (**Figures 2C, D**). Furthermore, results for western blot assays also showed that after overexpression of miR-125a, the expression level of mesenchymal marker N-cadherin was significantly downregulated, whereas a moderate elevation was observed in E-cadherin, which is a well-defined epithelial marker, indicating that miR-125a was a suppressor for EMT in NPC cells. Accordingly, migration marker MMP9 and VM marker LAMB2 was simultaneously decreased after miR-125a transfection, suggesting the inhibitory role of miR-125a on NPC migration and VM formation (**Figure 2E**). These results demonstrated that low level of miR-125a triggered formation of VM and may play a vital role in tumor progression in NPCs.

### miR-125a Targets TAZ in NPC

**Figure 3:**
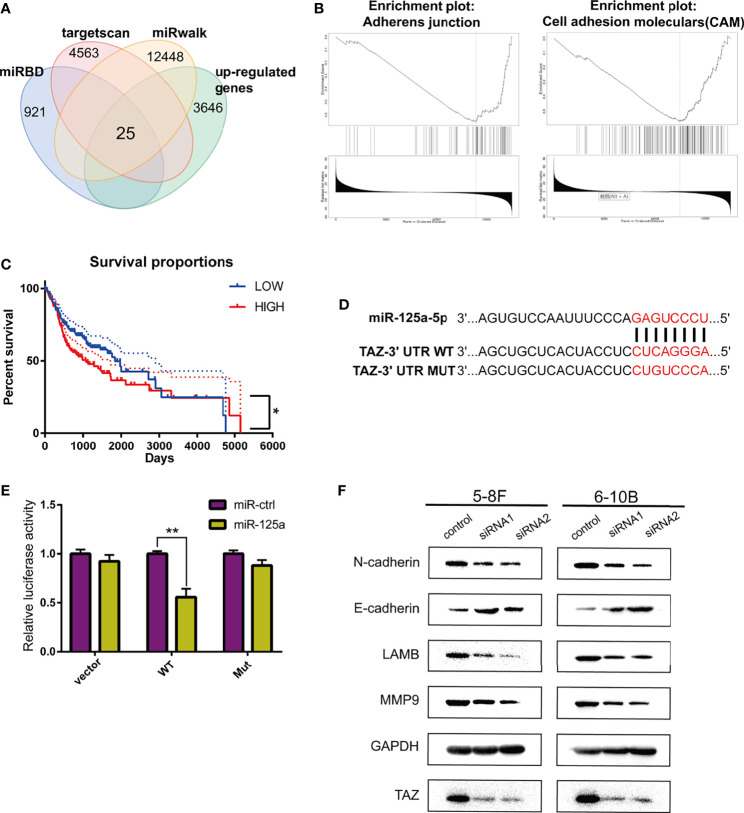
TAZ is the downstream target of miR-125a. **(A)** Venn diagram shows peak overlapping of the four factors. **(B)** GSEA for TAZ in HNSCC based on TCGA database. **(C)** Kaplan–Meier analysis in HNSCC patients. **(D)** Predicted binding sites and the designed mutant versions for miR-125a in the TAZ 3’-UTR. **(E)** Luciferase activity was decreased when miR-125a was overexpressed in 293T cells. **(F)** Western blot assay detecting TAZ and EMT-related proteins. (*P < 0.05; **P < 0.01).

To determine the target of miR-125a in NPC, we firstly detected the differentially expressed genes using TargetScan (http://www.targetscan.org/), miRwalk (http://mirwalk.umm.uni-heidelberg.de/), miRDB (http://mirdb.org/) and the TCGA database for prediction, and 25 genes were selected (**Figure 3A**).

We subsequently identified the candidate target-genes according to the following criteria: (1) metastasis-related genes in NPC based on LinkedOmics (http://www.linkedomics.org/) (25); and (2) had the definite function of tumor suppressor based on literature review. Finally we selected TAZ to be a potential target of miR-125a. TAZ (tafazzin) is negatively associated to adherens junction and cell adhension moleculars according to the Gene Set Enrichment Analysis (**Figure 3B**), which indicated that TAZ has the positive correlation with metastasis.

Kaplan–Meier survival curves in HNSCC were constructed using the TCGA clinical data, and low TAZ level were prognostic of better overall survival in HNSCC patients (**Figure 3C**). TAZ activation is widespread in many human tumors, and closely related to EMT progression (26). The luciferase activity in miR-125a-transfected 293T cells decreased significantly, which demonstrated that miR-125ap directly targeted TAZ (**Figures 3D, E** **)**.

Furthermore, the effects of the knockdown of TAZ resembled the effects of miR-125a overexpression, which decreased the protein levels of E-cadherin, LAMB, MMP9, and N-cadherin (**Figure 3F**).

### miR-125a Inhibits Migration and VM Formation *via* TAZ

To verify that TAZ plays a crucial role along the tumor-suppressive biological progression of miR-125a, we performed a rescue experiment by over-expressing both miR-125a and TAZ in NPC cells.

**Figure 4:**
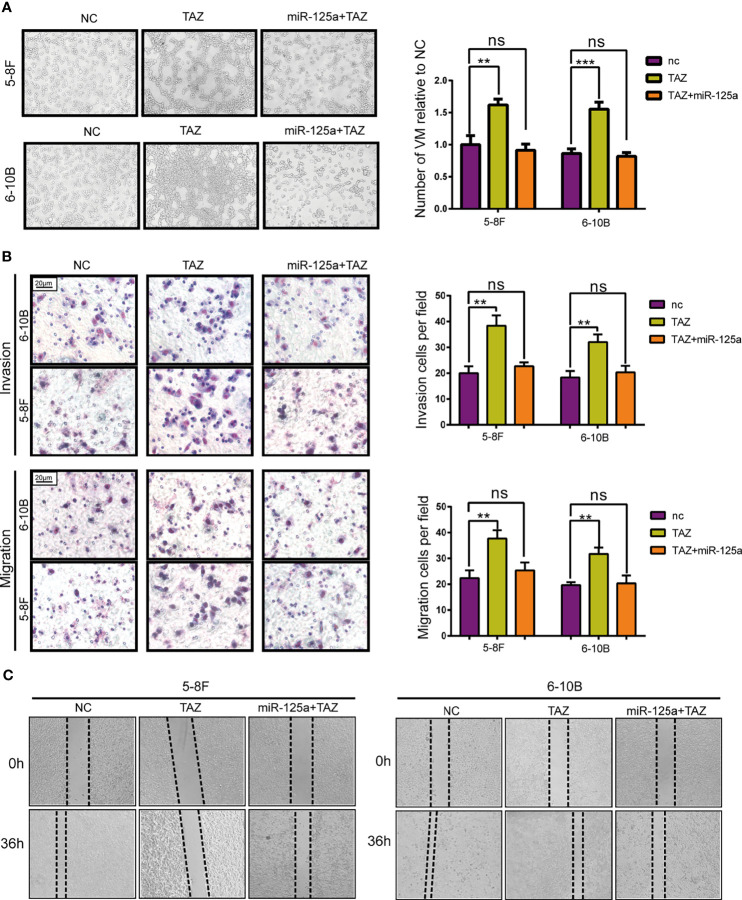
TAZ mediates the tumor suppressing function of miR-125a. **(A)** VM formation assay (left panel) and bar graph showing relative number of VM (right panel). **(B)** Invasion and migration transwell assay and histogram showing cell counting per field. **(C)** Wound healing assay. (ns, not significant; **P < 0.01; ***P < 0.001).

After rescuing the expression of TAZ, the number of VM tunnel reduced compared to that in group of TAZ-high expression (**Figure 4A**). To further confirm the opposing effects of miR-125a and TAZ in NPC cells, we conducted transwell assay (**Figure 4B**) and wound healing assay (**Figure 4C**). The results indicated that invasion and migration showed a significant reduction when co-transfecting miR-125a and TAZ in NPC cells.

Altogether, these results show that TAZ has pro-metastasis roles, which expression was directly suppressed by miR-125a.

### Transfected MSCs Packaging miR-125a Into Exosomes Which Can Suppress NPC Cells

As the MSCs are known to package miRs into exosomes (18), we hypothesized that after transducting miR-125a in MSC, MSCs would package miR-125a into exosomes and release these exosomes (EXO-miR-125a), which is of therapeutic importance.

**Figure 5:**
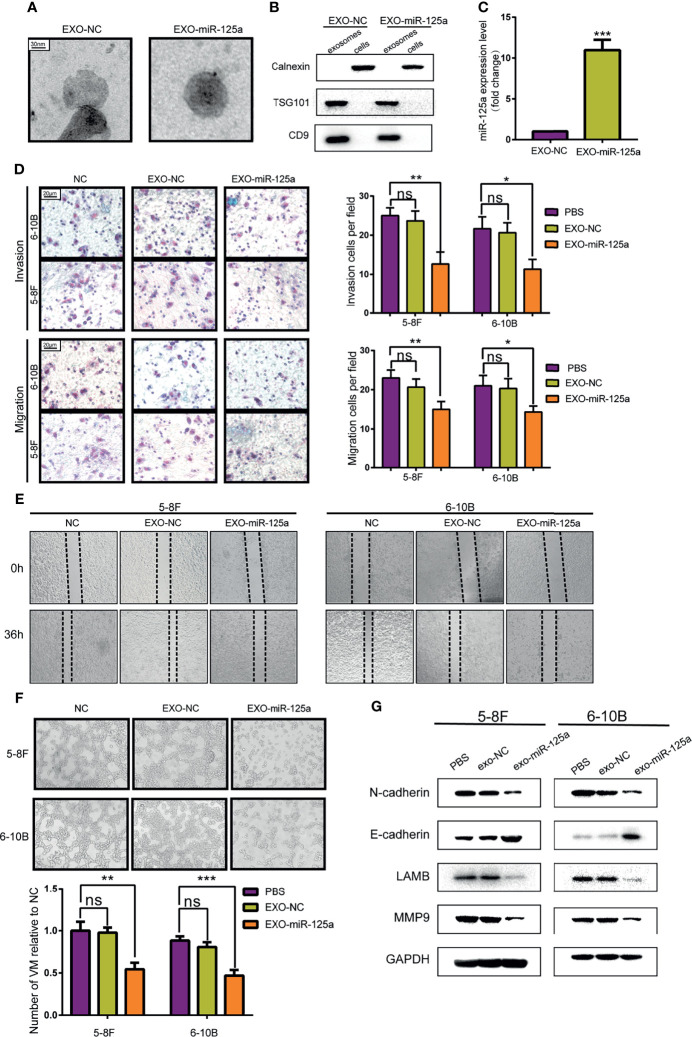
Anti-tumor effect of MSCs-exosomes overexpressing miR-125a *in vitro*. **(A)** Electron microscopic photographs of MSC-derived exosomes. **(B)** Western blot analysis showing the presence for TSG101 and CD9 and the absence of calnexin in exosomes. **(C)** miR-125a expression in NPCs detected by qRT-PCR after treatment with EXO-NC or EXO-miR-125a. **(D)** Transwell assay with histogram for cell numbers. **(E)** Wounding assay detected at 0 and 36 h respectively. **(F)** VM formation assay with quantified chart. **(G)** Western blot assay detecting EMT-related proteins. (ns, not significant; *P < 0.05; **P < 0.01; ***P < 0.001).

To validate the hypothesis, MSCs was transfected with miR-125a or scramble vector, and then exosomes were harvested from the medium and detected by electron microscopy (**Figure 5A**). Western blot verified further that the nanoparticles contained the exosome markers TSG101 and CD9 but absent for endoplasmic membrane marker calnexin (**Figure 5B**). The expression level of miR-125a in MSC exosomes was confirmed by PCR, and the level of miR-125a was increased after transfection (**Figure 5C**).

Transwell assays and wounding assays were performed for NPC cells pre-treated with exosomes for 48 h. The results indicated that EXO-miR-125a decreased migration of NPC cells (**Figures 5D, E** **)**. The VM formation capacity was reduced by miR-125a-transfected MSC exosomes (**Figure 5F**), which is in accordance with the VM inhibitory capability of miR-125a. Furthermore, the results of western blot showed that EXO-miR-125a decreased the invasion markers (N-cadherin and MMP9) and VM formation marker LAMB2 in 5–8F and 6–10B cell lines (**Figure 5G**).

### miR-125a *via* Exhibits Therapeutic Efficacy in Treating Xenograft Model

**Figure 6:**
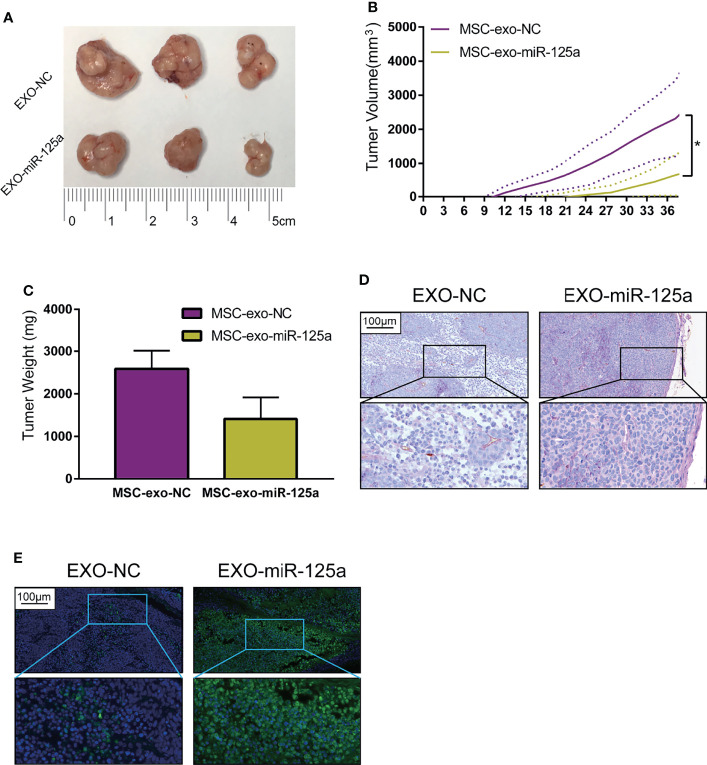
EXO-miR-125a suppressed tumor growth and VM formation *in vivo*. Mice were subcutaneously injected with 6–10B cells, and treated by exosomes. **(B)** Tumor volumes were monitored every 3 days. **(C)** After sacrifice, tumor weights were measured. **(D)** Representative images of the tumor HE staining and CD34-PAS staining of VM structures. **(E)** Representative images of tumor sections processed in the TUNEL assay (*P < 0.05).

To extend our findings *in vivo*, we established the mouse subcutaneous xenograft model of 6–10B cells (**Figure 6A**). Xenografted mice were randomized to receive EXO-NC or EXO-miR-125a treatment.

The tumor burden of mice in the EXO-miR-125a group was much smaller than that in EXO-NC group (**Figure 6B**), and similarly, the solid tumor weight taken at day of sacrifice was lower (**Figure 6C**).

Moreover, CD34/PAS staining indicated that EXO-miR-125a treatment hampered the VM formation abilities (**Figure 6D**), and TUNEL assay demonstrated that EXO-miR-125a enhanced the level of apoptosis of tumor cells. These results suggesting that exosomal miR-125a, plays a role in the anti-tumor efficacy *in vivo*.

## Discussion

Tumor growth depends closely on the formation of new blood vessels for their supply of oxygen and nutrients through not only angiogenesis (27), which is a promosing target for anti-tumor therapy. Several clinical trials for anti-angiogenic therapy have been evaluated in the field of NPC (28), focusing on VEGF inhibitor Bevacizumab or Sunitinib inhibiting vascular formation. However, despite the tumor shrinking in part of patients, the studies have been discontinued due to the severe side effects such as hemorrhaging. Besides, VEGF inhibition has been shown to induce hypoxic micro-environment which reduces therapeutic effectiveness (29). These evidences show that anti-VEGF therapy remain suboptimal. Additionally, some other pathways of vessel formation may exist in NPC that are able to bypass VEGF-promoted angiogenesis.

VM, defined by PAS^+^/CD34^−^ staining, are tube-like structures that imitate the function of endothelial blood vessels. VM has been described in NPC (30) and associated with rapid disease progression and poor patient outcomes (10). Epstein–Barr virus (EBV), plays a crucial role in NPC malignant behaviors, which is also associated with VM formation. Cells infected with EBV readily form the VM structures were evident in NPC and EBVaGC biopsies (8, 31). Evidence supports that VM structures promote tumor dissemination and metastasis (32). Furthermore, the formation of VM is strongly associated with the abnormal expression of several miRs, suggesting that miRs are potential targets for anti-VM therapy (9).

In this study, we revealed that miR-125a, which is a tumor suppressor in various malignant tumors (33), attenuates migration and VM formation in NPCs. Farias et al. found that VM formation was associated with a poor prognosis in NPC (34), which is consistent with the results in this study. We also found that VM formation is increased by low-level of miR-125 targeting TAZ, a key functional component of the Hippo pathway (35), which plays vital roles in tumor proliferation and invasion. Based on the findings above, we further provided information to clarify the role of miR-125a and TAZ in VM structure formation and NPC pathophysiology, which may be of great importance for NPC therapy (36). Other molecular components in Hippo-pathway besides TAZ may also be contributory and deserve further study.

The use of MSCs as delivery vehicles has been well studied in different diseases (37). It has been reported that MSCs could be used for anti-tumor agents delivery (38). However, intravenous administrated MSCs are possibly trapped in lungs due to their large size (39). Considering this limitation, MSC-derived exosomes could be alternatively utilized for anti-tumor agents delivery (40). The use of miR-carrying exosomes in NPC therapy remains relatively novel and unexplored. Extracellular vesicles such as exosomes are naturally released from MSCs (20). In current study, we artificially engineered human MSC by upregulating miR-125a and collected the exosome. Intriguingly, we found that the exosome contained large amount of miR-125a, and treating NPCs with miR-125a-carrying exosomes inhibits VM formation both *in vitro* and *in vivo*. The study did have limitations in our technical capabilities. First, preclinical models such as organoids were not used, which recapitulate structural and functional aspects of tumor precisely *in vivo* (41, 42). Second, further studies are awaited investigating the relationship between EBV infection with VM formation. Overall, our result provides strong evidence for the translational feasibility and efficacy of a therapeutically delivery mechanism, exosomal miR-125a.

## Data Available Statement

The original contributions presented in the study are included in the article/**Supplementary Material**. Further inquiries can be directed to the corresponding author.

## Ethics Statement

The animal study was reviewed and approved by the ethics committee of Fudan University.

## Author Contributions

JL, LK, and FW conceived and designed the experiments. FW, LC, and SG performed the experiments. HZ, JH, and LK analyzed the data. FW and JL wrote the paper. All authors listed have made a substantial, direct, and intellectual contribution to the work and approved it for publication.

## Funding

This work was supported by the National Key Research and Development Program of China (project No. 2018YFC0115700), the Science and Technology Commission of Shanghai Municipality (project No. 19411951000), the Science and Technology Development Fund of Shanghai Pudong New Area (No. PKJ2020-Y53) and the Shanghai Sailing Program (No. 20YF1428000).

## Conflict of Interest

The authors declare that the research was conducted in the absence of any commercial or financial relationships that could be construed as a potential conflict of interest.

## Publisher’s Note

All claims expressed in this article are solely those of the authors and do not necessarily represent those of their affiliated organizations, or those of the publisher, the editors and the reviewers. Any product that may be evaluated in this article, or claim that may be made by its manufacturer, is not guaranteed or endorsed by the publisher.

## Supplementary Material

Supplementary Figure 1 Protein level after knockdown or upregulation miR-125a. **(A)** Expression of TAZ reduced after up-regulated the miR-125a level, and increased after knocking down miR-125a. **(B)** Rescue assay. The function on EMT-related markers of high-level of miR-125a was offset by over-expressing of TAZ
