# Supplementary figures and images for: Mesenchymal Stem Cells Inhibits Migration and Vasculogenic Mimicry in Nasopharyngeal Carcinoma Via Exosomal MiR-125a
Source: Front Oncol. 2022 Feb 17;12:781979. doi: 10.3389/fonc.2022.781979 (PMC8892602; doi:10.3389/fonc.2022.781979)

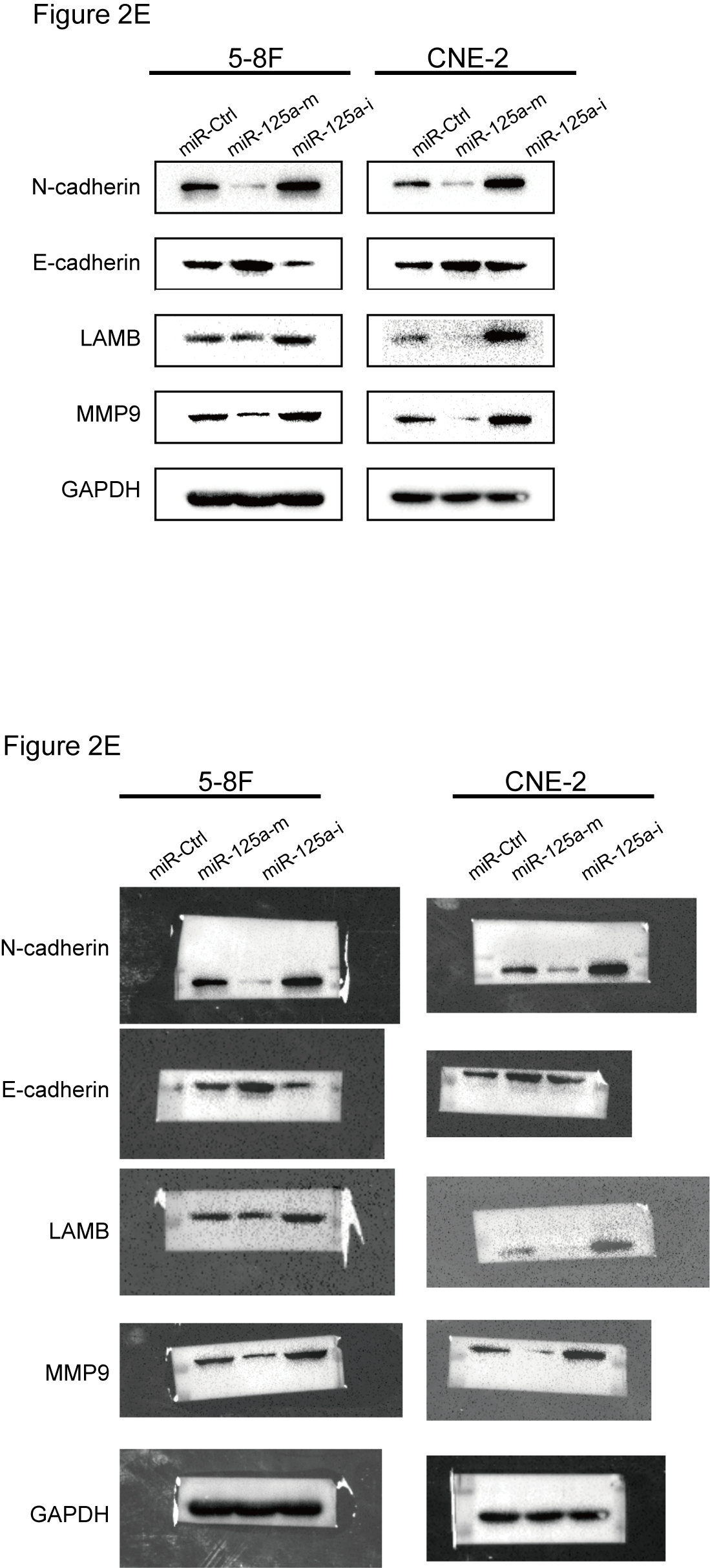

Supplement: Supplementary Figure 1 — Protein level after knockdown or upregulation miR-125a. (A) Expression of TAZ reduced after up-regulated the miR-125a level, and increased after knocking down miR-125a. (B) Rescue assay. The function on EMT-related markers of high-level of miR-125a was offset by over-expressing of TAZ [file DataSheet_5.zip › Figure 2E.TIF]

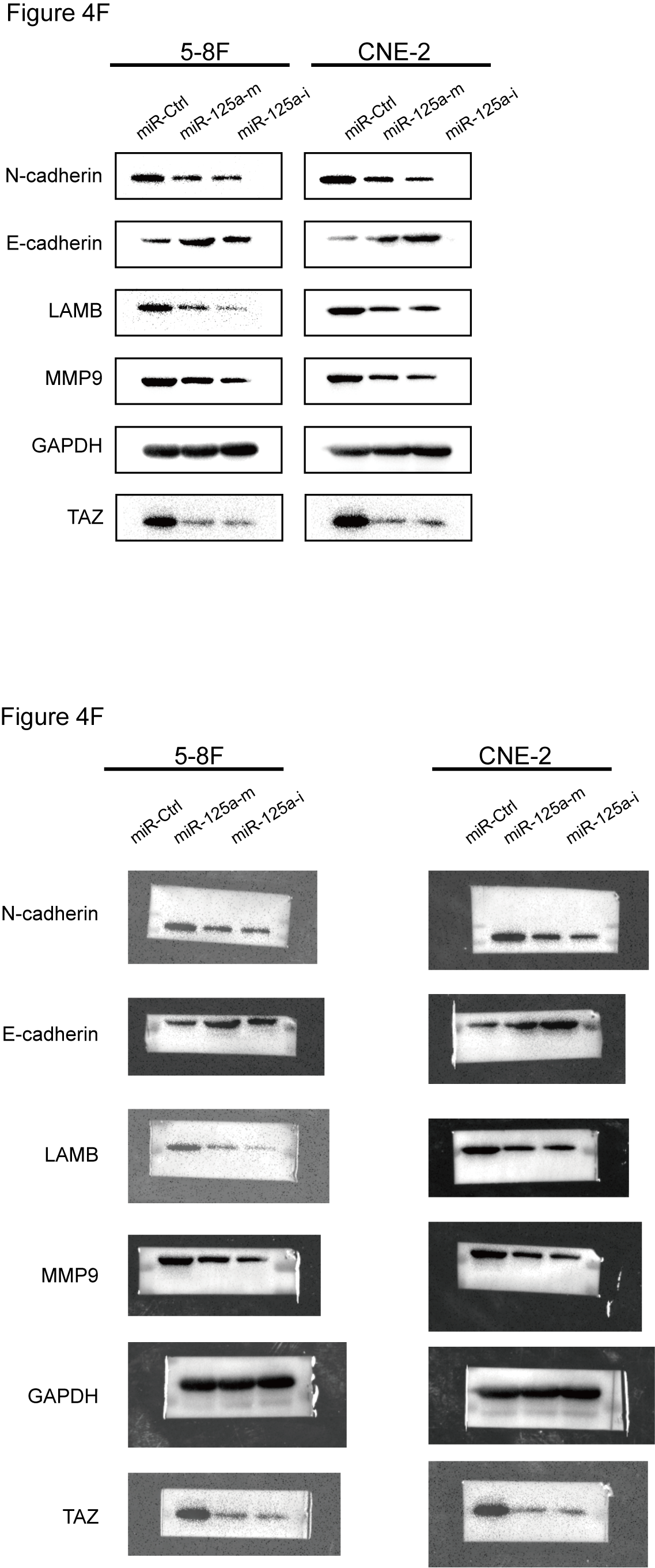

Supplement: Supplementary Figure 1 — Protein level after knockdown or upregulation miR-125a. (A) Expression of TAZ reduced after up-regulated the miR-125a level, and increased after knocking down miR-125a. (B) Rescue assay. The function on EMT-related markers of high-level of miR-125a was offset by over-expressing of TAZ [file DataSheet_5.zip › Figure 4 F.TIF]

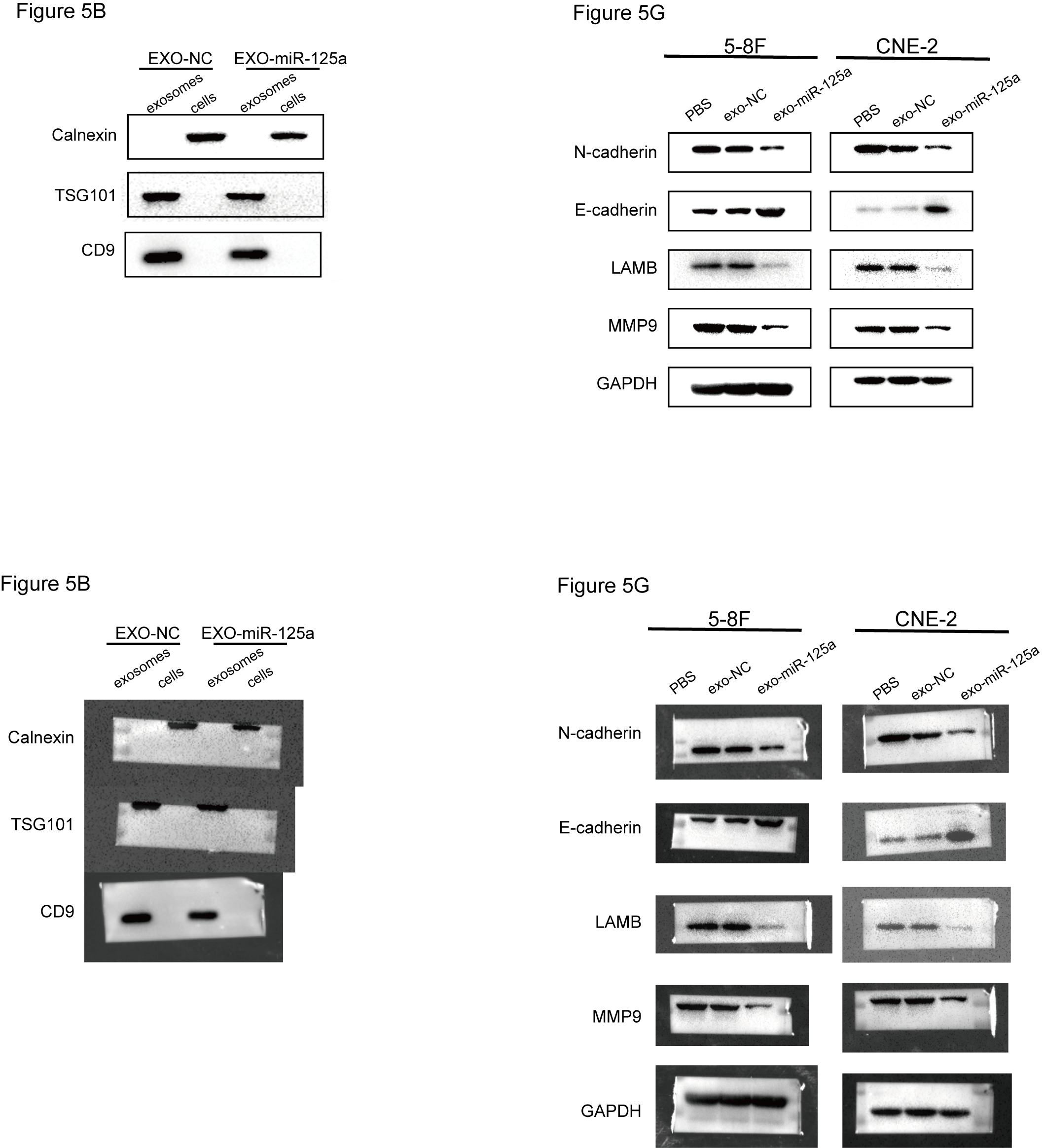

Supplement: Supplementary Figure 1 — Protein level after knockdown or upregulation miR-125a. (A) Expression of TAZ reduced after up-regulated the miR-125a level, and increased after knocking down miR-125a. (B) Rescue assay. The function on EMT-related markers of high-level of miR-125a was offset by over-expressing of TAZ [file DataSheet_5.zip › Figure 5B, G.TIF]

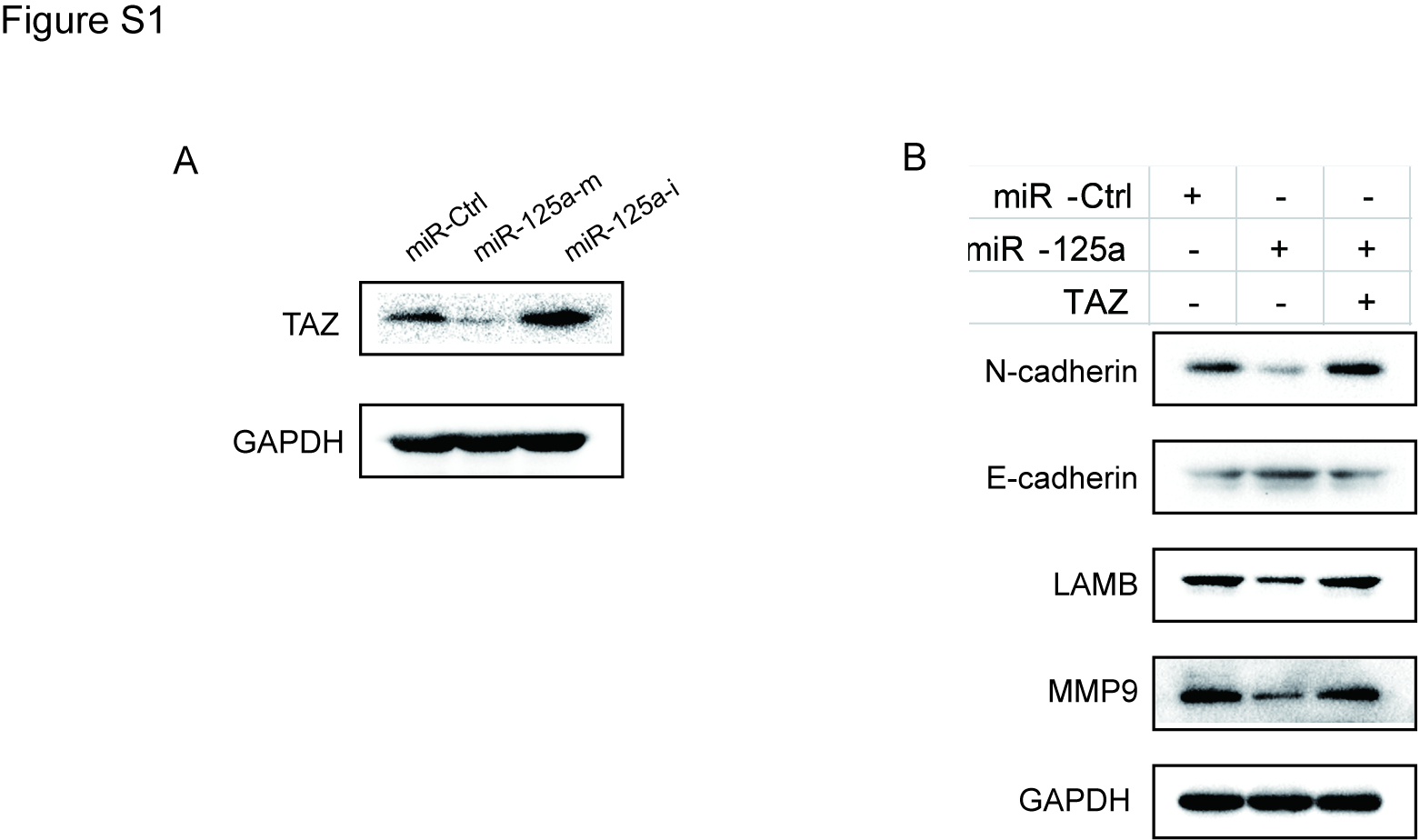

Supplement: Supplementary Figure 1 — Protein level after knockdown or upregulation miR-125a. (A) Expression of TAZ reduced after up-regulated the miR-125a level, and increased after knocking down miR-125a. (B) Rescue assay. The function on EMT-related markers of high-level of miR-125a was offset by over-expressing of TAZ [file DataSheet_5.zip › Supplementary Figure 1.TIF]
